# Bacteriocins from *Limosilactobacillus* and *Ligilactobacillus*: ecological logic, mechanistic diversity, and translational potential in the post-*Lactobacillus* taxonomy

**DOI:** 10.3389/fmicb.2026.1875597

**Published:** 2026-07-15

**Authors:** Samson Baranzan Wayah, Kensuke Arakawa, Koshy Philip

**Affiliations:** 1 Graduate School of Environmental, Life, Natural Science, and Technology, Okayama University, Okayama, Japan; 2 Department of Biochemistry, Faculty of Life Sciences, Kaduna State University, Kaduna, Nigeria; 3 Institute of Ocean and Earth Sciences, University of Malaya, Kuala Lumpur, Malaysia; 4 Faculty of Medicine, Nursing and Health Sciences, SEGi University, Kuala Lumpur, Malaysia

**Keywords:** bacteriocins, lactic acid bacteria, *Ligilactobacillus*, *Limosilactobacillus*, microbial ecology

## Abstract

Bacteriocins are ribosomally synthesized antimicrobial peptides that contribute to microbial competition, niche establishment, and community structure. The 2020 taxonomic reorganization of the former broad *Lactobacillus* genus provides a useful framework for reinterpreting bacteriocin diversity in lineage-specific ecological contexts. This review focuses on bacteriocins produced by species now assigned to *Limosilactobacillus* and *Ligilactobacillus*, two host- and food-associated genera that include several reported bacteriocin producers. These genera were selected because they contain bacteriocins with diverse structural features, including cyclic peptides, class IIa and IIb peptides, class IId peptides, defensin-like peptides, and larger proteinaceous bacteriocins, and because many producer strains originate from competitive ecological niches such as the gastrointestinal tract, oral cavity, vagina, milk, poultry, livestock, and fermented foods. We synthesize evidence on bacteriocin biosynthetic gene clusters, molecular diversity, antimicrobial mechanisms, ecological functions, physicochemical stability, and translational potential. This review also distinguishes bacteriocin-specific evidence from effects attributable to bacteriocin-producing strains, particularly for immunomodulation, co-aggregation, pathogen exclusion, and microbiota modulation. Finally, we address how comparative genomics, structured genome mining and artificial intelligence-aided prediction can speed bacteriocin discovery, emphasizing the necessity of experimental validation, standardized activity assays, safety evaluation and scalable production. This study gives a systematic framework to understand the bacteriocins of *Limosilactobacillus* and *Ligilactobacillus* in the post-*Lactobacillus* era by integrating taxonomy, ecology, mechanism and translational evidence.

## Introduction

Overuse and misuse of antibiotics in medicine, veterinary care, and farming are two key causes of antimicrobial resistance, which is still a major world health problem (Allel et al., 2023; Tang et al., 2023). In 2019, it was reported that bacterial antibiotic resistance was linked to about 4.95 million deaths around the world. Of these, 1.27 million deaths were directly linked to illnesses caused by resistant bacteria (Murray et al., 2022). It has been projected that antimicrobial resistance could contribute to 10 million deaths annually by 2050 (Nanayakkara et al., 2021). This number comes from scenario-based predictions and should be seen as a warning of what the future might bring rather than an exact prediction. In parallel with the medical pressure created by antimicrobial resistance, the food industry is scrambling to meet the rising demand for minimally processed foods that are preserved using antimicrobial techniques drawn from natural sources, as opposed to synthetic chemical preservatives (Chauhan and Rao, 2024). Due to these interrelated factors, bacteriocins are being considered more seriously as potential antimicrobials for use in agriculture, medicine, and the food industry (Jin Ng et al., 2020).

Bacteriocins are microbial peptides that are synthesized by the ribosomes (Cotter et al., 2013). These molecules assist bacteria to establish their niche (Bakkeren et al., 2025), to become competitive to facilitate access to restricted resources and to impact structure of the ecosystem in a good manner (Heilbronner et al., 2021). In addition to antibacterial activity, bacteriocins can also inhibit the growth of virus [enterocin CRL35 (Umair et al., 2022), Enterocin ST4V (Al Kassaa et al., 2014)], fungi [EntV (Graham et al., 2017; Cruz et al., 2022)], protozoa [enterocin as-48 (Abengózar et al., 2017), AdDLP (Huang et al., 2021)], and cancer cells [nisin (Giurini et al., 2024), enterocin LNS18 (Molujin et al., 2022)]. Their ability to kill mainly microorganisms that are phylogenetically or niche-related to them (Rutter et al., 2024) prompted their strong consideration, as potential antimicrobials for biopreservation of foods (Jurburg et al., 2022), modulation of microbiota (Heilbronner et al., 2021), and substitutes to antibiotics (Cotter et al., 2013). Lactic acid bacteria (LAB) capable of producing bacteriocin are given more attention over other bacteriocin-producing microorganisms (Chikindas et al., 2018). This is because their safety and ecological importance in foods that are fermented and environments within their hosts has been established many years ago (Liang et al., 2025).

The genus *Lactobacillus*, a wide genus, was recently reclassified (Zheng et al., 2020). This reclassification has dramatically transformed our view of the evolution and diversity of lactic acid bacteria. The separation of this genus into several phylogenetically related genera has brought to light, traits that are lineage-specific, but were previously obscure due to taxonomic overgeneralization. In particular, the *Limosilactobacillus* (Kabuki et al., 1997; Daniele et al., 2014; Wayah and Philip, 2018a) and *Ligilactobacillus* (Ocaña et al., 1999; Flynn et al., 2002; Messaoudi et al., 2012) genera have emerged as rich reservoirs of bacteriocins. These genera are characterized by unique ecological adaptations (; O'Shea et al., 2013; Abramov et al., 2022), genome structures (Zheng et al., 2020), and bacteriocin biosynthetic gene clusters (Kawai et al., 2001; O'Shea et al., 2013; Sevillano et al., 2023). This reclassification gives an important framework for reinterpreting previous bacteriocin investigations and for connecting genetic organization to ecological and functional outputs.

The previous *Lactobacillus* genus was split into many genera (Zheng et al., 2020), but this review is focused on *Limosilactobacillus* and *Ligilactobacillus*. This focus does not suggest that production of bacteriocins, niche adaptability or antibacterial specificity are restricted to these taxa. Instead, these two genera were selected because they contain many bacteriocin-producing species and strains that have been reported from host- and food-associated settings. These environments include the gastrointestinal tract (Sevillano et al., 2023), oral cavity (), vagina (Ocaña et al., 1999), milk (Mahdi et al., 2016), poultry (Svetoch et al., 2011), livestock (Wayah and Philip, 2018a), and fermented foods (Shan et al., 2023). They therefore provide a useful case study for examining how post-*Lactobacillus* taxonomy can be used to reinterpret bacteriocin diversity, ecological function, and translational potential.

Other reclassified lactobacilli, including members of *Lactiplantibacillus* (Rocchetti et al., 2021; Li et al., 2023), *Lacticaseibacillus* (Wei et al., 2022; Xue et al., 2024), *Levilactobacillus* (Somashekaraiah et al., 2021; Iliev et al., 2025), *Lentilactobacillus* (Appel et al., 2025), and related genera (Wang et al., 2021), also produce important bacteriocins. It is necessary to treat these groups independently due to the fact that they have different distribution within the ecosystem and genetic origins. Also, they have different bacteriocin repertoires. The aim of this review is therefore not to claim exclusivity for *Limosilactobacillus* and *Ligilactobacillus*, but to evaluate the evidence available for bacteriocins from these two lineages within a clearly defined taxonomic and ecological framework. Reported bacteriocins from *Ligilactobacillus* and *Limosilactobacillus* have various structural and functional traits. Some are modified after translation (Kawai et al., 2001; Sevillano et al., 2023) while others are not modified after translation (Flynn et al., 2002; Stern et al., 2006). These features are not unique to these two genera. However, their distribution across host- and food-associated lineages gives an informative basis for comparing bacteriocin structure, ecological context, and potential application.

Their biosynthetic gene clusters are responsible for producing the core peptides, proteins needed to modify peptides after translation, and proteins that protect the producer strain from being killed by its bacteriocin. Also, these clusters of genes produce proteins involved in regulation of bacteriocin production, maturation, and release outside the cell of the producer (Flynn et al., 2002; Sevillano et al., 2023). Moreover, this type of genetic organization is aided by horizontal gene transfer (Sharp and Foster, 2025), gene duplication (Sharp and Foster, 2025), and selective pressures as a consequence of competition among microorganisms (Veening and Blokesch, 2017; Heilbronner et al., 2021). This helps to create various gene clusters that are functionally specialized and specific to selected lineages.

Bacteriocins also have an effect on the environment by changing the population dynamics (Arbulu and Kjos, 2024; Hugerth et al., 2024) and endurance of the microbe community in complex groups (Sommer et al., 2017). Bacteriocin production can make host-associated and food fermentation microbiome ecosystems more competitive. Due to this, they become less likely to be occupied by other microorganisms (Auchtung et al., 2025). Also, some bacteriocins do not greatly alter the makeup of the microbial community of their producer (Snyder et al., 2024). The emergence of mechanisms of bacteriocin immunity and resistance (Draper et al., 2015; Soltani et al., 2021) shows how important systems-level evaluations that combine ecological context with molecular processes are. Progress in computational biology genomics aids research on bacteriocin (Damoczi et al., 2024; Seshadri et al., 2025). High-throughput sequencing has facilitated comparative analysis of bacteriocin biosynthetic gene clusters on a large scale (Dinglasan et al., 2025; Hourigan et al., 2025). Moreover, new artificial intelligence (AI)-aided approaches may speed up bacteriocin discovery and development. The use of machine-learning and deep learning in genome mining, structure-function prediction, and data-driven peptide engineering is becoming popular (Ma et al., 2022; Zhang et al., 2023; Hamamsy et al., 2024). These new methods offer fresh opportunities to speed up discovery of bacteriocins and improve their characteristics (Hemmerling and Piel, 2022). Combining these approaches with classical microbiology such as activation of silent genes (Rutledge and Challis, 2015) and systems biology (Theuretzbacher et al., 2023) can reposition bacteriocins as precision antimicrobials.

In this review, we synthesize current information on *Limosilactobacillus* and *Ligilactobacillus*-derived bacteriocins framing their biosynthetic gene cluster, diversity, modes of action, and ecological functions within the context of the 2020 taxonomic reorganization. We show how comparative genomics and AI-powered strategies are changing discovery and design of bacteriocins. Also, we discuss translational avenues and future directions for harnessing the full potential of bacteriocins for food and medical applications. By combining taxonomy, ecology, evolution, and emergent computational approaches, this review aims to give a coherent framework for understanding and extending the frontiers of bacteriocin research in the post-*Lactobacillus* taxonomy.

## Literature-search strategy, scope of the review, and tools used

This review was prepared as a structured narrative review focused on bacteriocins produced by species currently assigned to *Limosilactobacillus* and *Ligilactobacillus*. Literature searches were conducted between November 2025 and January 2026 using PubMed, Scopus, Web of Science, Google Scholar, and ScienceDirect. The search terms included combinations of former and current taxonomic names and bacteriocin-related keywords: “*Lactobacillus* bacteriocin,” “*Limosilactobacillus* bacteriocin,” “*Ligilactobacillus* bacteriocin,” “*Lactobacillus* salivaricin,” “*Ligilactobacillus* salivaricin,” “*Lactobacillus* fermencin,” “*Limosilactobacillus* fermencin,” “*Lactobacillus* reutericin,” and “*Limosilactobacillus* reutericin.” Reference lists of relevant primary studies and reviews were also manually screened to identify additional reports.

Studies were included when they reported bacteriocins or bacteriocin-like inhibitory substances from species now classified as *Limosilactobacillus* or *Ligilactobacillus*, especially where purification, molecular mass or sequence information, biosynthetic gene-cluster evidence, antimicrobial mechanism, food-model testing, or *in vivo* evidence was available. Studies were excluded when antimicrobial activity was attributed only to non-bacteriocin metabolites, when the producer organism could not be assigned taxonomically, when the study focused on unrelated lactic acid bacterial genera, or when review articles did not provide primary evidence relevant to the reviewed bacteriocins. Titles and abstracts were screened by authors, and full texts were examined for studies meeting the inclusion criteria. No language restriction was applied, although accessible full-text information and extractable experimental details determined whether a study could be incorporated into the evidence synthesis.

Because many bacteriocins were originally reported before the 2020 reclassification of the former *Lactobacillus* genus, this review reports the current taxonomic name where possible and provides the former name used in the original publication to preserve traceability to the primary literature. The review is not intended to cover all bacteriocin-producing genera derived from the former *Lactobacillus* genus. Instead, it focuses on *Limosilactobacillus* and *Ligilactobacillus* as two ecologically informative lineages containing multiple bacteriocin-producing strains from host-associated and food-associated environments.

Figures were prepared using Figure Labs, including graphical layout/design functions. The authors provided the scientific content, checked all labels and mechanisms against the cited literature, and take full responsibility for the accuracy of the final figures.

## Diversity and reclassification of bacteriocin-producing lactobacilli

**Figure 1:**
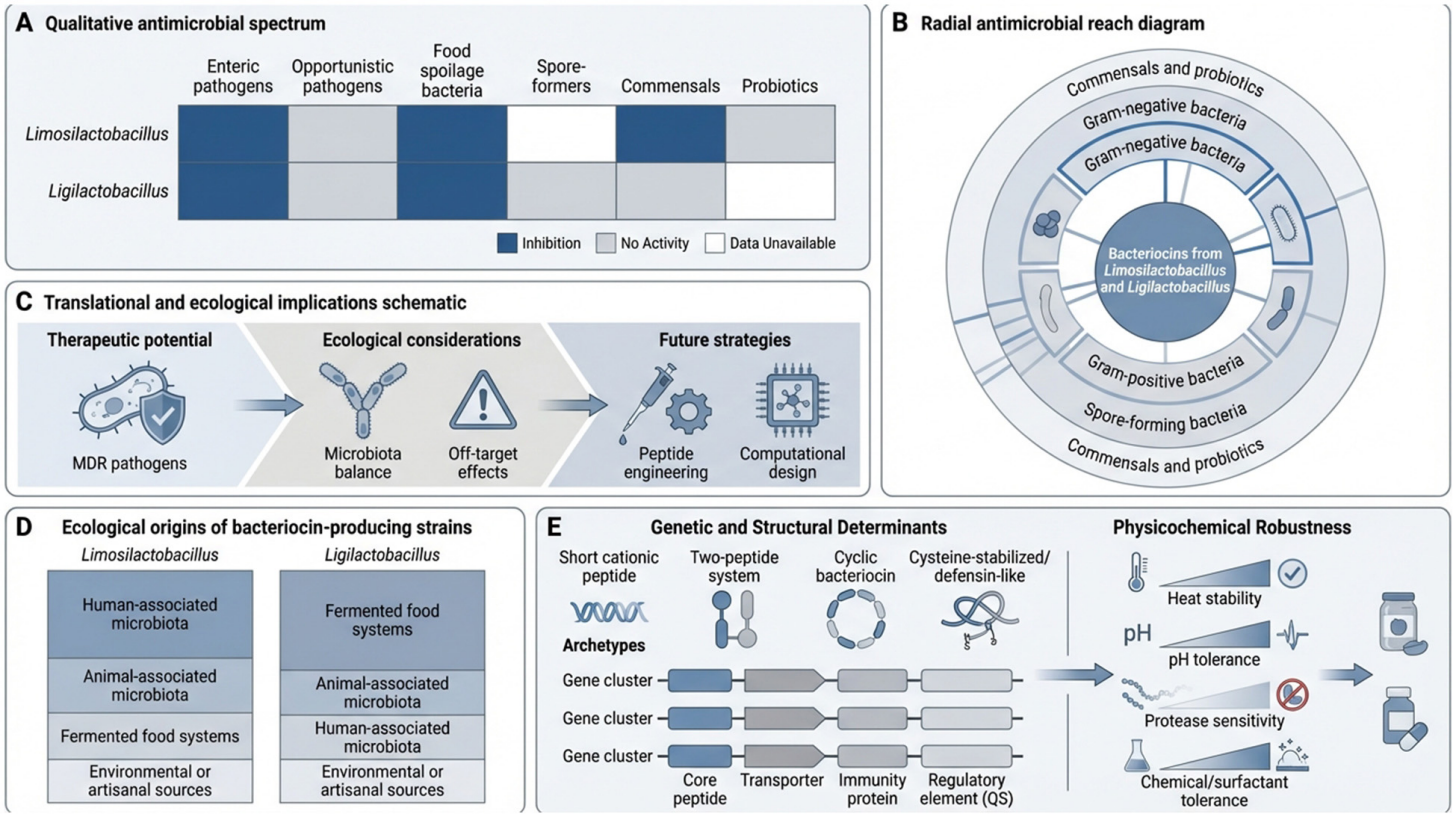
Diversity, ecological context, and translational evidence for bacteriocins from *Limosilactobacillus* and *Ligilactobacillus*. **(A)** Reported inhibitory spectra of selected bacteriocins or bacteriocin-containing preparations. Activity against Gram-positive bacteria is generally better supported, whereas Gram-negative activity should be interpreted in relation to purification status, assay conditions, and co-treatments. **(B)** Microbiota-related effects reported for selected bacteriocin systems or bacteriocin-producing strains. These effects are not universal and require strain-level or bacteriocin-specific validation. **(C)** Potential applications in food biopreservation, veterinary use, and therapeutic development. **(D)** Ecological niches from which bacteriocin-producing strains have been isolated, including gastrointestinal, oral, vaginal, milk, poultry, livestock, and fermented-food environments. **(E)** Genetic, structural, and physicochemical features influencing bacteriocin function, including biosynthetic gene clusters, peptide class, molecular mass, charge, hydrophobicity, disulfide bonds, cyclic structure, thermostability, pH stability, and protease sensitivity.

The 2020 reclassification of the former broad *Lactobacillus* genus clarified phylogenetic relationships among lactobacilli and created an opportunity to reinterpret older bacteriocin studies using current taxonomy (Zheng et al., 2020). Within this framework, *Limosilactobacillus* and *Ligilactobacillus* are useful focal genera because they include several bacteriocin-producing strains isolated from host-associated and food-associated niches. Examples include reutericin 6 from *Limosilactobacillus reuteri* formerly reported as *Lactobacillus reuteri* (Kawai et al., 2001), fermencin SA715 from *Limosilactobacillus fermentum* formerly reported as *Lactobacillus fermentum* (Wayah and Philip, 2018a), Abp118 from *Ligilactobacillus salivarius* formerly reported as *Lactobacillus salivarius* (Flynn et al., 2002; Corr et al., 2007), and bactofencin A from *Ligilactobacillus salivarius* (O'Shea et al., 2013). These examples do not represent all bacteriocins from reclassified lactobacilli, but they illustrate how current taxonomy can improve comparison across producer lineages, ecological niches, and antimicrobial phenotypes. The ecological niches of *Limosilactobacillus* and *Ligilactobacillus* include fermented foods (Rasheed et al., 2020; Halder and Mandal, 2025), various parts of their hosts [saliva (Busarcevic et al., 2008), Busarcevic and Dalgalarrondo, 2012, (Wannun et al., 2016), milk (Wayah and Philip, 2018a; Abramov et al., 2023), vagina (Ocaña et al., 1999; Pascual et al., 2008)] and gastrointestinal tract (Toba et al., 1991; Flynn et al., 2002; Stern et al., 2006; Svetoch et al., 2011; Messaoudi et al., 2012; O'Shea et al., 2013; Therdtatha et al., 2016; Wayah and Philip, 2018b; Li et al., 2021; Sevillano et al., 2023; Elnar et al., 2025) (Figure 1D). Bacteriocin-producing *Limosilactobacillus* and *Ligilactobacillus* are predominantly found in humans (Ocaña et al., 1999; Flynn et al., 2002), animals (Sevillano et al., 2023; Yoo et al., 2023) and poultry (Stern et al., 2006; Messaoudi et al., 2012; Li et al., 2021; Elnar et al., 2025). The niches they occupy within these hosts are characterized by high microbial competition for nutrients (Raffatellu, 2018) which drives development of various antimicrobial approaches (Kommineni et al., 2015; Sugrue et al., 2024).

The broad ecological distribution of bacteriocin-producing *Limosilactobacillus* and *Ligilactobacillus* likely contributes to the diversity of bacteriocins reported from these genera (Palmer and Foster, 2022). However, ecological distribution alone does not determine bacteriocin diversity. Other factors such as horizontal gene transfer (Hourigan et al., 2025), plasmid carriage (Miceli de Farias et al., 2024), gene loss (Heilbronner et al., 2021), duplication (Ness et al., 2014), regulatory variation (Arbulu and Kjos, 2024), and strain-specific selection (Sugrue et al., 2024) also shape bacteriocin repertoires. This diversity is also reflected in their varied regulatory mechanisms and architecture comprising plasmid (such as salivaricin P) (Elnar et al., 2025) or chromosome-borne operons (for example Abp 118) (Flynn et al., 2002), and quorum-sensing strategies (Flynn et al., 2002) which reveals the strong association between phylogeny, ecological niche and antimicrobial role.

Framing bacteriocin diversity within the reorganized *lactobacillus* genus gives a strong analytical and conceptual basis for unraveling their function, evolution and potential applications. Analyzing bacteriocins within the reclassified taxonomy facilitates interpretation of their biosynthetic gene cluster within the context of common evolutionary histories. Due to vertical inheritance, closely related taxonomic groups (such as *Limosilactobacillus* and *Ligilactobacillus*) often have similar bacteriocin biosynthetic gene cluster (Egan et al., 2018) while niche competition drives horizontal gen transfer, culminating in variations (Zheng et al., 2015; Brito, 2021).

With this approach, it is easier to draw direct lines between bacteriocin structure and inhibitory spectrum. Genus or niche-specific targets (such as fermented food, vaginal, and intestinal microflora) of bacteriocins are often related with their structural attributes such as receptor-binding motifs, post-translational changes, length, and distribution of charge (Palmer and Foster, 2022; Sugrue et al., 2024). Consequently, reclassification enhances understanding of the reasons why some bacteriocins exhibit narrow and ecologically selective antimicrobial activity (Rea et al., 2010; Mills et al., 2017) while others display wider inhibitory spectrum (Lozo et al., 2017; O'Connor et al., 2018).

Situating bacteriocins within the reorganized taxonomic group sheds light on their ecologic selectivity. Bacteriocins do not randomly kill microorganisms instead, they are antimicrobial weapons which selectively kill other microorganisms within their niche providing competitive edge to the producer (Acedo et al., 2018). Taxonomy-guided evaluations uncovers the relationship between production of bacteriocin, host, availability of nutrients, and structure of microbial community, indicating their use in stabilizing the niche rather than random killing (Hols et al., 2019).

This framework helps to identify potential applications of the bacteriocins. Targeted bacteriocin discovery for food biopreservation, therapeutic and probiotic applications is enhanced by establishing relationship between taxonomy, biosynthetic gene cluster, and antimicrobial spectrum. Researchers can focus on taxonomic groups having high ecological pressure and bacteriocin diversity that are suited for specific use instead of relying only on empirical screening. Using this model, the mechanisms of antimicrobial action, physicochemical attributes, and translational challenges of bacteriocins produced by *Limosilactobacillus* and *Ligilactobacillus* can be seamlessly compared.

## Evidence levels used in this review

The evidence base for bacteriocins from *Limosilactobacillus* and *Ligilactobacillus* is heterogeneous. Some bacteriocins, such as Abp118 (Flynn et al., 2002), bactofencin A (O'Shea et al., 2013), reutericin 6 (Kawai et al., 2001), salivaricin variants (Pingitore et al., 2009; O'Shea et al., 2011; Wayah and Philip, 2018b), and nisin S (Sevillano et al., 2023), have sequence, structural, genetic, or mechanistic information. Others are supported mainly by partial purification (Mahdi et al., 2016), molecular-mass estimation (Mahdi et al., 2019), protease sensitivity, or inhibition assays (Mahdi et al., 2016; Elnar et al., 2025). To avoid overinterpretation, this review distinguishes among: bacteriocin-specific evidence, where purified peptides (O'Shea et al., 2011; Wayah and Philip, 2018a), defined gene clusters (O'Shea et al., 2013), mutant analysis (Corr et al., 2007), Riboulet-Bisson et al, 2012, or heterologous expression (Mesa-Pereira et al., 2017) support attribution to a bacteriocin; preparation-level evidence, where activity is shown using partially purified fractions or cell-free supernatants; and strain-level evidence, where inhibition, immunomodulation, co-aggregation, or microbiota modulation is demonstrated for the producing organism but not necessarily for the bacteriocin molecule alone (Table 1).

**Table 1:** Evidence summary for bacteriocins reported from species currently assigned to *Limosilactobacillus* and *Ligilactobacillus*.

Current producer species	Former name in original report	Strain	Bacteriocin	Source/ niche	Class / structural type	Molecular mass/ sequence	Purification/ evidence status	Main targets	Activity data (as reported)	BGC/ genetic evidence	Application evidence	Reference
*Limosilactobacillus*												
*Limosilactobacillus fermentum*	*Lactobacillus fermentum*	L23	Bacteriocin L23	Human vagina	Putative class II	<7,000 Da	Partially purified; protease-sensitive	Broad spectrum including Gram-positive, Gram-negative, and *Candida* spp.	640 AU/mL; inhibition zones reported for multiple targets	Not reported	*In vitro* only: proposed potential vaginal application	(Pascual et al., 2008)
*Limosilactobacillus fermentum*	*Lactobacillus fermentum*	GA715	Fermencin SA715	Goat milk	Novel; non-pore-forming, cell wall-associated	1792.537 Da	Purified to homogeneity; MALDI-TOF confirmed; protease-sensitive	Broad spectrum including Gram-positive and Gram-negative bacteria	MIC: 8.93–103.57 μM depending on target	Not reported	Food-model and scale-up evidence: extended banana shelf life	Wayah and Philip, 2018a
*Limosilactobacillus fermentum*	*Lactobacillus fermentum*	3872	BLF3872	Human breast milk	Class III bacteriocin; bacteriolysin	Predicted ~ 42.5 kDa	Genome-identified; function supported at strain/supernatant level	*Staphylococcus aureus* and selected Gram-negative pathogens	6-log reduction of *S. aureus* in 24 h; 3–4 log reduction of *Escherichia. coli, Salmonella*; ATP leakage reported	Genome sequencing + BAGEL + comparative sequence analysis support class III bacteriocin assignment	*In vitro*/*ex vivo* evidence: immunomodulatory strain-level effects reported	Abramov et al., 2022, 2023
*Limosilactobacillus reuteri*	*Lactobacillus reuteri*	LA6	Reutericin 6	Human infant feces	Class I circular bacteriocin identical to gassericin A	5,652 Da and primary structure identical to gassericin A	Purified to homogeneity; structurally re-evaluated by HPLC/MS/ sequencing	Mainly *Lactobacillus* spp.	160 AU/mL maximum activity	PCR + sequencing showed that LA6 carries the exact structural gene for gassericin A; gaa/reu plasmid genes later shown to be identical to those of gassericin A producers	*In vitro* only: potentially useful in treatment of dental carries, animal diarrhea, mastitis, and as a potential food biopreservative since it is identical to gassericin A.	Toba et al., 1991; Kawai et al., 2001; Arakawa et al., 2010; Pandey et al., 2013; Hu et al., 2018; Liang et al., 2021
*Limosilactobacillus reuteri*	*Lactobacillus reuteri*	9	Reutericin_LHS	Human breast milk	Not reported	Estimated: 10,600 Da	Partially purified; protease-sensitive	*Shigella flexneri, Salmonella enterica*	ZOI: 27.7 mm and 23.5 mm, respectively	Not reported	*In vitro* evidence: potentially has *in vivo* inhibitory activity against *Shigella flexneri* and *Salmonella enterica*	Mahdi et al., 2016
*Limosilactobacillus panis*	*Lactobacillus panis*	C-M2	Lactocin C-M2	Fermented corn flour	Not reported	863.52 Da	Purified to homogeneity; LC -MS/MS confirmed	Broad spectrum including Gram-positive and Gram-negative bacteria	ZOI: <10–> 18 mm	Not reported	Food-model evidence: extended shelf life of fresh fish when used with dielectric barrier discharged cold plasma	Shan et al., 2023
*Ligilactobacillus*												
*Ligilactobacillus salivarius*	*Lactobacillus salivarius*	UCC118	Abp118	Human GIT	IIb: Abp118α and Abp118β	4096.69 Da	Purified to homogeneity; MS confirmed	*Listeria innocua, Listeria monocytogenes, Bacillus coagulans*.	51,600 AU/mL	DNA sequencing identified Abp 118 locus comprising of 18 ORF	*In vivo* evidence: protected mice against infection by *Listeria monocytgenes*; showed intestinal microbiome-sparing ability	Flynn et al., 2002; Corr et al., 2007, Riboulet-Bisson et al, 2012
*Ligilactobacillus salivarius*	*Lactobacillus salivarius*	CGMCC 2070	Bacteriocin XJS01	Intestine of Yunnan black-bone chicken	Not reported	666.31 Da	Purified to homogeneity; LC -MS/MS confirmed	*Staphylococcus aureus, Shigella flexneri*	MIC: 9.85 μg/mL, 18.75 μg/mL, respectively	Not reported	Food-model evidence: extended shelf life of raw pork loins and beef	Ying et al., 2023; Ng et al., 2025
*Ligilactobacillus salivarius*	*Lactobacillus salivarius*	NRRLB-30514	Bacteriocin OR-7	Caecum of broiler chicken	IIa	5,123 Da	Purified to homogeneity; MALDI-TOF confirmed; protease-sensitive	*Campylobacter jejuni*	Significant Log reduction in *C. jejuni*	Not reported	*In vivo* evidence: significantly reduced number of *C. jejuni* in chicken ceca	Stern et al., 2006
*Ligilactobacillus salivarius*	*Lactobacillus salivarius*	1077	Bacteriocin L-1077	Caecum of broiler chicken	IIa	3,454 Da	Purified to homogeneity; MALDI-TOF confirmed; protease-sensitive	Broad spectrum including Gram-positive and Gram-negative bacteria	MIC: 0.09–1.5 μg/mL depending on target	Not reported	*In vivo* evidence: significantly reduced number of *C. jejuni* and *Salmonella* Enteritidis in chicken ceca, liver and spleen	Svetoch et al., 2011
*Ligilactobacillus salivarius*	*Lactobacillus salivarius*	SMXD 51	Bacteriocin SMXD51	Caecum of chicken	Not reported	5383.2 Da	Purified to homogeneity; MALDI-TOF confirmed	Broad spectrum including Gram-positive and Gram-negative bacteria Including *Campylobacter jejuni*	ZOI: <3–6 mm	Not reported	*In vitro* evidence: potentially reduced *C. jejuni* infection in chicken via direct inhibition and immunomodulation	Messaoudi et al., 2012; Saint-Cyr et al., 2017
*Ligilactobacillus salivarius*	*Lactobacillus salivarius*	BGH O1	Bacteriocin LS2	Human oral environment	IId	4115.1 Da	Purified to homogeneity; ESI-TOF confirmed	Broad spectrum including *Shigella* spp. and *Yersinia* spp.	ZOI: 1–8 mm	DNA sequencing identified Bacteriocin LS2 locus	*In vitro*: proposed potential control of oral pathogens	Busarcevic et al., 2008, Busarcevic and Dalgalarrondo, 2012
*Ligilactobacillus salivarius*	*Lactobacillus salivarius*	CRL1328	Salivaricin CRL 1328	Human vagina	IIb: Salα and Salβ	Predicted: 4096.1 and 4333.1 Da, respectively	Partially purified and Chemically synthetized	*Enterococcus faecalis*	MIC: 1.5 μM	DNA sequencing identified gene cluster	*In vitro*: proposed potential control of vaginal infections	Ocaña et al., 1999; Pingitore et al., 2009, Vera Pingitore et al, 2009
*Ligilactobacillus salivarius*	*Lactobacillus salivarius*	K4	Sal K and Alb β	Chicken intestine	IIb	4346.18 and 4448.11 Da, respectively	Chemically synthesized	*Streptococcus* sp., *Staphylococcus aureus, Enterococcus faecalis*	MIC: 3.59 μM	Not reported	*In vitro*: proposed potential poultry application	Pilasombut et al., 2005; Sangtanoo et al., 2014; Thongkhao et al., 2018
*Ligilactobacillus salivarius*	*Lactobacillus salivarius*	DPC 6488	Salivaricin L	Neonatal isolate	IId	4117 Da	Purified to homogeneity; MALDI-TOF confirmed	*Listeria innocua, Listeria monocytogenes*	MIC_50_: 20 μM	DNA sequencing identified gene cluster	*In vitro*: proposed potential human therapeutic	O'Shea et al., 2011
*Ligilactobacillus salivarius*	*Lactobacillus salivarius*	DPC 6488	Salivaricin T	Neonatal isolate	IIb: SalTα, SalTβ	5656.3, 5270.5 Da, respectively	Purified to homogeneity; MALDI-TOF confirmed	*Lactobacillus*	Not reported	DNA sequencing identified gene cluster	*In vitro*: proposed potential human therapeutic	O'Shea et al., 2011, 2012
*Ligilactobacillus salivarius*	*Lactobacillus salivarius*	KL-D4	Salivaricin KLD	Duck intestine	Putative: class IIb	Predicted: 4,202 Da	Purified to homogeneity;	Broad spectrum including Gram-positive and Gram-negative bacteria	100–25,600 AU/mL	PCR + DNA sequencing identified gene cluster	Food-model evidence: preserved creamy filling	Therdtatha et al., 2016
*Ligilactobacillus salivarius*	*Lactobacillus salivarius*	Not reported	Salivaricin LHM	Human saliva	Not reported	Estimated: 13,500 Da	Partial purification	*Pseudomonas aeruginosa*	MIC: 16–128 mg/mL	Not reported	*In vivo* evidence: significantly reduced number of *P. aeruginosa* in mice via direct killing and probably immunomodulation	Mahdi et al., 2019
*Ligilactobacillus salivarius*	*Lactobacillus salivarius*	DPC6502	Bactofencin A	Porcine intestine	IId	2782.7 Da	Purified to homogeneity; MALDI-TOF confirmed	*Staphylococcus aureus, Listeria monocytogenes*	MIC: 1–5 μM	Genome sequencing identified bactofencin A locus	*In vitro* evidence: subtle impact on gut microbiota; non-toxic and showed intestinal instability	O'Shea et al., 2013; O'Connor et al., 2018; Bédard et al., 2019; Soltani et al., 2022; O'Connor et al., 2025
*Ligilactobacillus salivarius*	Not applicable	B4112, B4311, B5258, B5121, L5301	Salivaricin P	Chicken intestine	IIb: salPα and β	Predicted: 4,097, 4,285 Da	Not purified: Cell-free supernatant	*Listeria monocytogenes, Enterococcus faecalis*	ZOI: 7.79–13.08 mm	Genome sequencing identified salivaricin P locus	*In vitro*: proposed potential poultry application	Elnar et al., 2025
*Ligilactobacillus salivarius*	Not applicable	P1CEA3	Nisin S	Pig intestine	I	3347.99 Da	Purified to homogeneity; MALDI-TOF confirmed	Gram-positive bacteria and *Escherichia coli*	ZOI: <5–>10 mm	Genome sequencing + comparative sequence analysis support class I bacteriocin assignment	*In vitro* only: proposed potential swine application	Sevillano et al., 2023
*Ligilactobacillus salivarius*	*Lactobacillus salivarius*	SPW1	Salivaricin mmaye1	Human GIT	Not reported	1221.074 Da	Purified to homogeneity; MALDI-TOF confirmed	*Micrococcus luteus, Streptococcus mutans, Staphylococcus aureus*	MIC: 3.44–6.88 μM	Not reported	*In vitro* only: proposed potential human therapeutic application	Wayah and Philip, 2018b
*Ligilactobacillus agilis*	Not applicable	C7	Agilicin C7	Pig faeces	IId	Estimated: 10,000 Da	Purified to homogeneity	*Listeria monocytogenes, Enterococcus faecium, Enterococcus faecalis*	ZOI: 1–5 mm	Genome sequencing identified the bacteriocin locus	*In vitro* only: proposed potential swine application	Yoo et al., 2023
*Ligilactobacillus agilis*	Not applicable	LDTM 47	Bacteriocin LDTM47	GIT of broiler chicken	IIa	Not reported	Not reported	*Listeria monocytogenes*	Not reported	Genome sequencing + comparative sequence analysis support class IIa bacteriocin assignment	*In vitro* and *in silico*: proposed potential food and application	Eum et al., 2025
*Ligilactobacillus ruminis*	*Lactobacillus ruminis*	Not reported	Lactocin DT1	Not reported	IIa	Not reported	Not reported	Enterococci	MID: 0.0035–0.7	Not reported	*In vitro only*: proposed potential treatment of enterococcal infections	Tresnak and Hackel, 2020
*Ligilactobacillus animalis*	*Lactobacillus animalis*	TSU4	Bacteriocin TSU4	GIT of Catla catla	Putative: II	4,117 Da	Purified to homogeneity; Q-TOF LC/MS confirmed	Broad spectrum including Gram-positive and Gram-negative bacteria	ZOI: 23–32 mm	Not reported	*In vitro*: proposed potential application in maintaining fish health; *in vivo* evidence: safe, non-toxic, non-immunogenic	Sahoo et al., 2015, 2017

GIT, gastrointestinal tract; ZOI, zone of inhibition; MID, minimum inhibitory dilution; MIC, minimum inhibitory concentration; PCR, polymerase chain reaction; BGC, biosynthetic gene cluster.

## Genetic and structural determinants of bacteriocin diversity

Bacteriocins from *Limosilactobacillus* and *Ligilactobacillus* have various structural characteristics including those with pediocin N-terminal motif [bacteriocin OR-7 (Stern et al., 2006), bacteriocin L-1077 (Svetoch et al., 2011), bacteriocin LDTM47 (Eum et al., 2025)], HXXXD and HXH motifs [bacteriocin BLF3872 (Abramov et al., 2023)], one-peptide liner system [bacteriocin LS2 Busarcevic and Dalgalarrondo, 2012, agilicin C7 (Yoo et al., 2023)], two-peptide linear system [Abp118 (Flynn et al., 2002), salivaricin CRL 1328 (Pingitore et al., 2009), salivaricin T (O'Shea et al., 2011), salivaricin P (Elnar et al., 2025), cyclic (reutericin 6 (Kawai et al., 2001)], cysteine-maintained structure [bacteriocin BLF3872 (Abramov et al., 2023), bactofencin A (O'Shea et al., 2013)], small cationic defensin-like [Bactofencin A (O'Shea et al., 2013)], and secondary structures such as α-helix, β-sheet, and turn [Sal K and Alb β (Sangtanoo et al., 2014; Thongkhao et al., 2018)]. Moreover, their biosynthetic gene clusters are chromosome or plasmid-borne and comprise of genes encoding core peptide, regulatory proteins, and transport proteins (Kawai et al., 2001; Flynn et al., 2002; Pingitore et al., 2009), Busarcevic and Dalgalarrondo, 2012. Regulation of their biosynthesis occur via quorum sensing (Pingitore et al., 2009; O'Shea et al., 2013), indicating their importance in microbial niche competition instead of unregulated production and release.

Physicochemical properties and antimicrobial activity of bacteriocins are influenced by their structural characteristics (Ekblad et al., 2016; Sugrue et al., 2024). These structural features include length, distribution of charge, disulfide linkages, and hydrophobicity (Sugrue et al., 2024). These genetic and structural features form the basis for unraveling the functions of various bacteriocins produced by *Limosilactobacillus* and *Ligilactobacillus* (Figure 1E).

## Evolutionary and ecological logic of bacteriocin diversification in reclassified lactobacilli

Understanding reasons behind the existence of structural diversity among bacteriocins produced by *Limosilactobacillus* and *Ligilactobacillus* requires exploration of their niches. These bacteriocin producers inhabit highly populated niches which have limited resources (Indo et al., 2021; Zhang et al., 2022). These include fermented foods (de Luna Freire et al., 2024; Mejía-Caballero and Marco, 2026), gastrointestinal tract (Montgomery et al., 2022; Hashimoto et al., 2025), milk (Quilodrán-Vega et al., 2020; Sun et al., 2025), vagina (Aragón et al., 2024), and oral cavity (Park et al., 2023) of humans, livestock and poultry (Table 1). In these ecological environments, ability to produce bacteriocins makes them more competitive (Gilbert and Lynch, 2019). Like many bacteriocins from other lactic acid bacteria and Gram-positive bacteria, several *Limosilactobacillus*- and *Ligilactobacillus*-derived bacteriocins show relatively selective activity, often against phylogenetically related or niche-sharing competitors Riboulet-Bisson et al, 2012. However, the degree of specificity varies by bacteriocin, target organism, assay system, and purification status (Table 1). Therefore, selectivity should be treated as a bacteriocin-specific property rather than a universal feature of these genera.

This ecological interpretation is supported by the structure of their bacteriocin biosynthetic gene clusters. Arrangement of genes encoding core peptides, regulatory proteins, immunity proteins and transporters in genetic modules that are exchangeable and can be easily rearranged, lost or gained enables quick evolutionary adaptation to high competition existing within their niches (Fedorec et al., 2021). Moreover, the fact the bacteriocin production is often regulated by quorum sensing reveals the influence of microbial density and ecology on bacteriocin biosynthesis (Hancock et al., 2025) thereby, supporting their biological function as antimicrobials with specificity that are produced only when needed not constitutively (Cornforth and Foster, 2013). The specificity and diversity in function observed among bacteriocins produced by *Limosilactobacillus* and *Ligilactobacillus* is explained by this evolutionary perspective.

## Mechanisms of antimicrobial action

**Figure 2:**
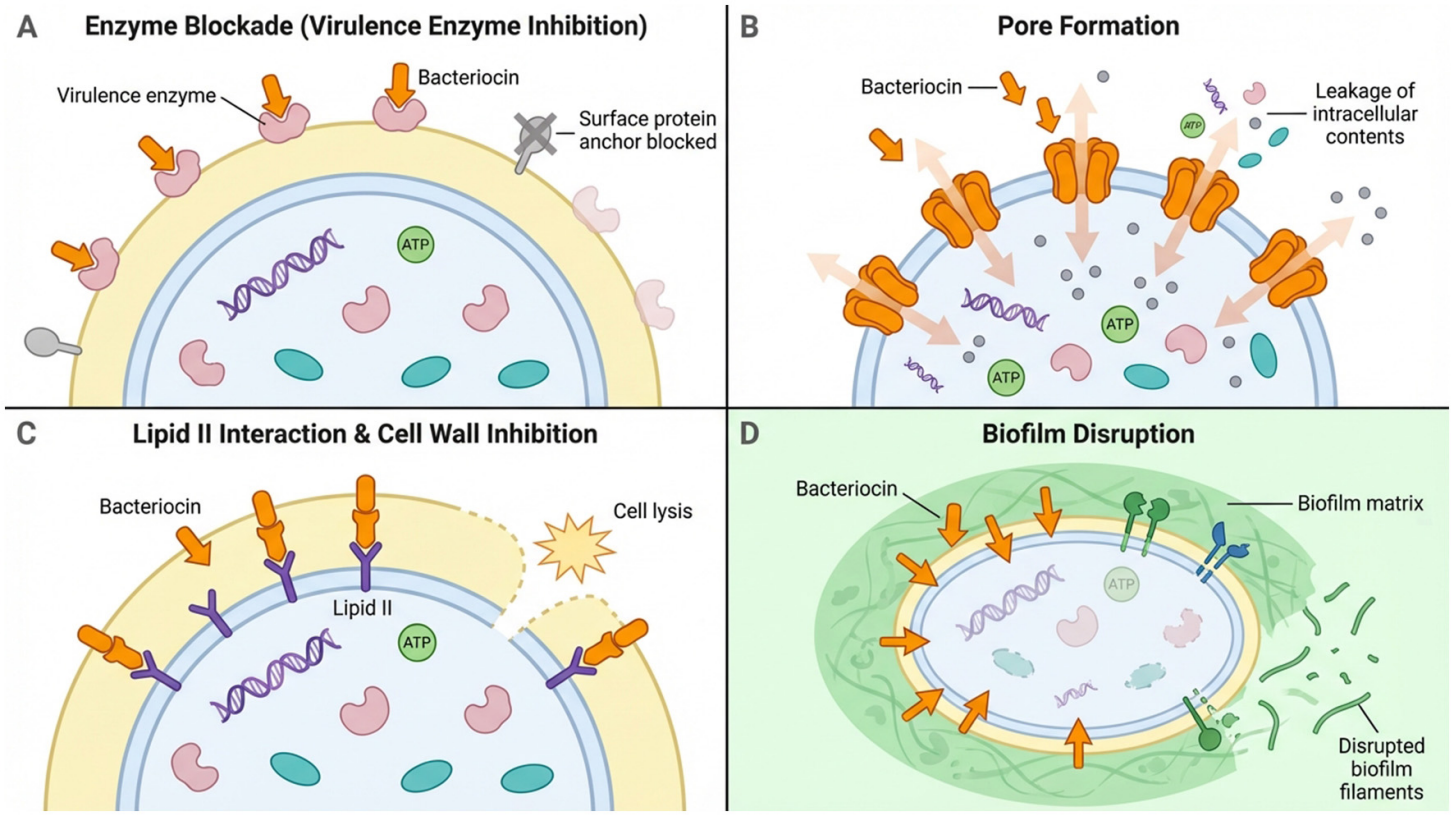
Reported and proposed mechanisms of action against Gram-positive bacteria. Mechanisms shown are not established for all bacteriocins from *Limosilactobacillus* and *Ligilactobacillus*. They summarize mechanisms reported for selected bacteriocins or inferred from related bacteriocin classes. **(A)** Binding to cell-envelope-associated targets, potentially reducing surface-protein anchoring or virulence-associated functions where supported by experimental evidence. **(B)** Membrane insertion and pore formation, causing depolarization and leakage of ATP, ions, nucleic acids, or proteins. **(C)** Interaction with lipid I/lipid II or related cell-wall biosynthesis components, leading to impaired peptidoglycan synthesis and cell-wall weakening. This mechanism should be assigned only to bacteriocins for which lipid II or cell-wall precursor interaction has been demonstrated or strongly inferred from class-specific evidence. **(D)** Antibiofilm effects, including impaired adhesion, biofilm disruption, and metabolic collapse. These effects may reflect purified bacteriocin activity or strain/preparation-level activity depending on the study.

**Figure 3:**
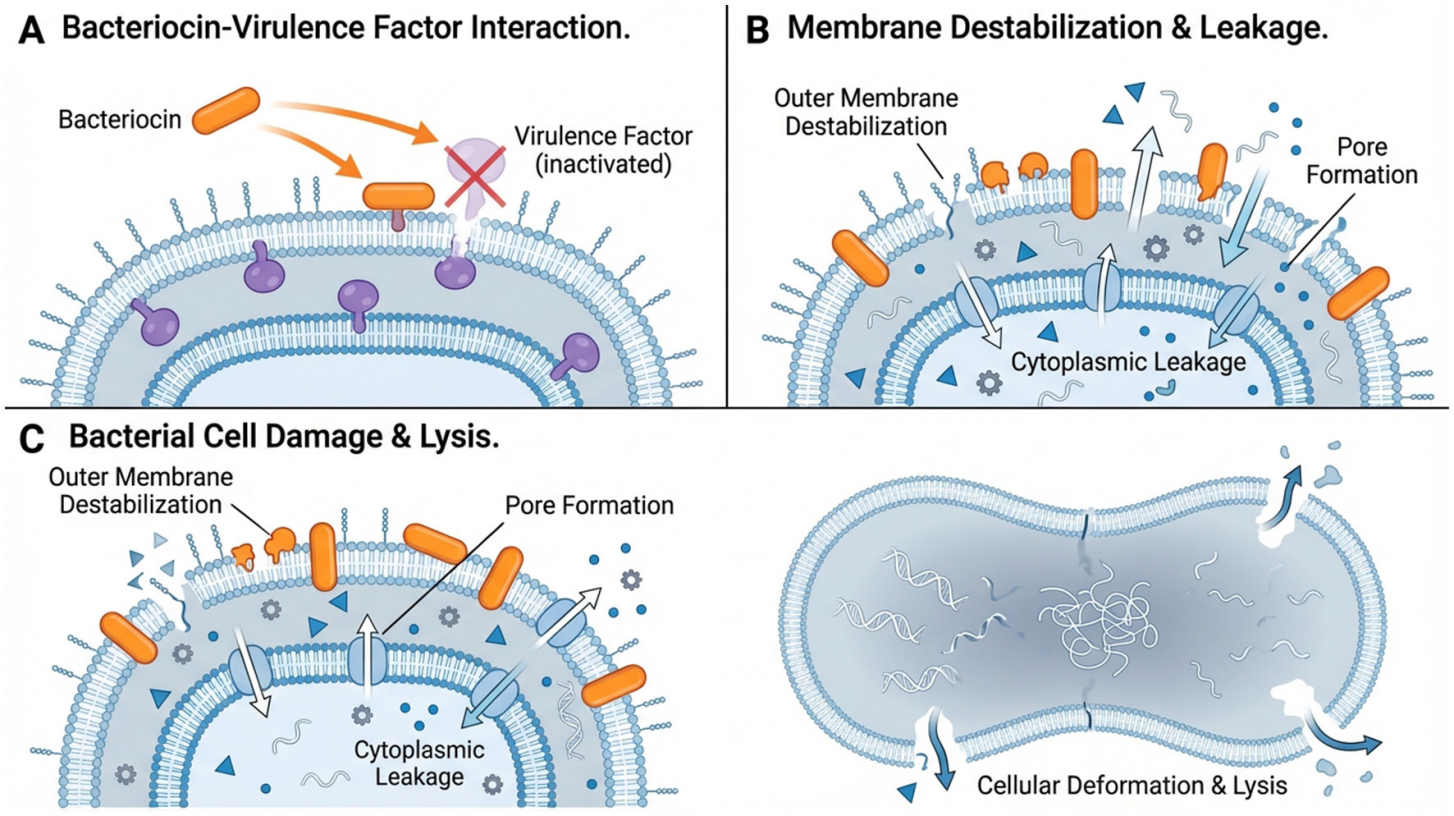
Conditional mechanisms reported against Gram-negative bacteria. Gram-negative bacteria are usually less susceptible to many lactic acid bacterial bacteriocins because the outer membrane limits access to inner-membrane and cell-wall targets. Therefore, the mechanisms shown should be interpreted as conditional and evidence-dependent. **(A)** Inactivation or binding of secreted or periplasmic virulence-associated factors, where experimentally supported. **(B)** Outer-membrane destabilization followed by inner-membrane permeabilization, pore formation, and leakage of intracellular contents. This activity may require co-treatments or stress conditions such as acidic pH, chelators, refrigeration, cold plasma, or food-processing hurdles. **(C)** Cellular deformation, including swelling, abnormal septation, cytoplasmic condensation, DNA disorganization, and lysis, as observed in selected microscopy-based studies.

Bacteriocins reported from *Limosilactobacillus* and *Ligilactobacillus* act through mechanisms that are broadly consistent with those described for bacteriocins from other lactic acid bacteria (Alvarez-Sieiro et al., 2016; Pang et al., 2022), although the level of mechanistic evidence varies considerably among individual peptides (Table 1). The best-supported antimicrobial effects involve damage to the bacterial cell envelope, including membrane permeabilization, pore formation, dissipation of membrane potential, and leakage of intracellular contents (Figures 2, 3). However, these mechanisms should not be generalized to all bacteriocins from the two genera because some studies were performed with purified peptides, whereas others relied on partially purified fractions, cell-free supernatants, or producer strains. Accordingly, mechanistic interpretation should be linked to the degree of bacteriocin characterization in each study.

In general, bacteriocins from lactic acid bacteria show stronger activity against Gram-positive bacteria than against Gram-negative bacteria (Helander et al., 1997; Chen et al., 2025). This difference is largely explained by cell-envelope architecture. Gram-positive bacteria expose the cytoplasmic membrane and cell-wall-associated targets more directly, whereas Gram-negative bacteria possess an outer membrane that restricts access to membrane-active peptides and cell-wall precursors (Field et al., 2018). Consistent with this principle, the evidence for *Limosilactobacillus*- and *Ligilactobacillus*-derived bacteriocins is strongest for Gram-positive targets, while reported activity against Gram-negative bacteria requires more cautious interpretation in relation to purification status, assay conditions, and the presence of co-treatments or other inhibitory factors (Figures 1A, 2, 3).

For Gram-positive bacteria, membrane disruption appears to be the most commonly supported mode of action. Several studies report increased membrane permeability (Bacteriocin XJS01, Salivaricin LHM, Sal K and Alb β) (Thongkhao et al., 2018; Mahdi et al., 2019; Li et al., 2021), pore formation (Bacteriocin XJS01, Salivaricin mmaye1, Lactocin CM-2) (Wayah and Philip, 2018b; Shan et al., 2020; Li et al., 2021), depolarization (Salivaricin CRL 1328, Sal K and Alb β) (Pingitore et al., 2009; Thongkhao et al., 2018), and leakage of ions and intracellular macromolecules (Bacteriocin BLF3872, Salivaricin LHM, Reutericin 6, Sal K and Alb β) (Kawai et al., 2004; Thongkhao et al., 2018; Mahdi et al., 2019; Abramov et al., 2022, 2023), after exposure to bacteriocins or bacteriocin-containing preparations from these genera (Figure 2B). For example, bacteriocin XJS01 from *Ligilactobacillus salivarius* formerly *Lactobacillus salivarius* showed bactericidal and antibiofilm activity against *Staphylococcus aureus*, with evidence of membrane damage and release of intracellular constituents (Li et al., 2021). Transcriptomic, proteomic and metabolomic analysis further suggested disruption of multiple cellular processes, including metabolism, stress response, adhesion, and cytolysis. This shows that membrane injury may be followed by broader physiological changes (Ying et al., 2023, 2024). Similarly, structural and functional studies on Salvicin K and the beta peptide from *Ligilactobacillus salivarius* K4 support a membrane-targeting mechanism consistent with rapid killing through loss of envelope integrity (Thongkhao et al., 2018).

Among the bacteriocins discussed in this article, Bactofencin A is one of the most described. Produced by *Ligilactobacillus salivarius* DPC6502, it is an unusual cationic, cysteine-containing class IId bacteriocin with a compact defensin-like architecture (O'Shea et al., 2013). Structure-activity analyses indicate that its C-terminal macrocycle is important for antimicrobial potency (O'Connor et al., 2018; Bédard et al., 2019), and experimental evidence supports a membrane-associated mode of action against susceptible Gram-positive targets, particularly *Staphylococcus aureus* (Figure 2B). Recent research has shown that death can be postponed in some situations, which raises the possibility that the experimental setting and target physiology determine the kinetics of bacteriocin-mediated damage (O'Connor et al., 2025). This observation highlights that killing of a bacterial target may not follow a uniform, immediate membrane-breakdown model even within one bacteriocin system.

In addition to direct membrane permeabilization, some bacteriocins may act through more specific interactions with cell-envelope-associated targets (Fermencin SA715, Sal K and Alb β) (Thongkhao et al., 2018; Wayah and Philip, 2018a). For post-translationally modified bacteriocins and lantibiotic-like systems, interference with lipid I or lipid II and the resulting inhibition of peptidoglycan biosynthesis is a plausible mechanism, as established for several bacteriocins outside these genera (Bauer and Dicks, 2005; Barreteau et al., 2009). However, direct demonstration of such target binding remains limited for most *Limosilactobacillus* and *Ligilactobacillus* bacteriocins reviewed here. This mechanism should therefore be assigned cautiously and mainly to peptides for which structural class or experimental evidence supports it, such as nisin S (Wiedemann et al., 2001), rather than inferred for the group as a whole (Figure 2C). Likewise, claims that bacteriocins from these genera bind surface-associated virulence determinants (Bacteriocin J32) (Heredia-Castro et al., 2021; Halder and Mandal, 2025) or intracellular targets involved in DNA replication or protein synthesis should be restricted to cases where such mechanisms have been directly investigated (Figure 2A).

Some bacteriocins or bacteriocin-containing preparations from *Limosilactobacillus* and *Ligilactobacillus* have also been reported to inhibit Gram-negative bacteria (Salivaricin LHM, Bacteriocin BLF3872, Bacteriocin LF-BZ532, Bacteriocin J32, Lactocin C-M2, Reutericin_LHS, Bacteriocin XJS01) (Mahdi et al., 2016, 2019; Rasheed et al., 2020; Shan et al., 2020; Heredia-Castro et al., 2021; Abramov et al., 2022; Jiang et al., 2022). However, these results need to be carefully interpreted. In many situations, other compounds or processes are used along side LAB bacteriocins to alter outer membrane of Gram-negative bacteria (Mokoena et al., 2021; Shan et al., 2023). Approaches used for this purpose include acidic conditions, chelators, food-processing stress, refrigeration, cold plasma, or other hurdle factors that enhance access to inner-membrane targets (Figure 3B). For instance, preservative effects involving bacteriocin-containing preparations from *Limosilactobacillus panis* C-M2 in aquatic foods were evaluated in combination with dielectric barrier discharge treatment (Shan et al., 2020). Therefore, it is improper to assign the full inhibitory effect to the bacteriocin alone. Similarly, it is not safe to assume that a pure bacteriocin may directly cross the Gram-negative outer membrane under normal circumstances just because a crude or partly purified product has shown broad-spectrum action. For this reason, the distinction between purified bacteriocin activity, preparation-level activity, and combined-treatment effects is especially important when interpreting Gram-negative inhibition (Figures 3A–C).

In addition to killing planktonic cells, some studies suggest bacteriocins can breakdown biofilms (Bacteriocin XJS01, Salivaricin LHM) (Mahdi et al., 2019; Jiang et al., 2022). One example is bacteriocin XJS01, which has been found to both harm the cell membrane and break *Staphylococcus aureus* biofilms (Li et al., 2021; Jiang et al., 2022). This reveals that antibiofilm effects may be caused by several processes. Such processes include direct killing, interference with adhesion, altered membrane physiology, and collapse of biofilm structure (Figure 2D). However, biofilms can also be inhibited when there is high acidity, competition for nutrients, or other effects associated with strains (Kim et al., 2026; Zhang et al., 2026). Therefore, as with other mechanistic claims, antibiofilm activity should be interpreted according to the experimental level at which it was demonstrated, whether with purified peptide, partially purified preparation, or bacteriocin-producing strain.

Taken together, the available evidence indicates that cell-envelope damage is the best-supported common antimicrobial outcome among the better-characterized bacteriocins from *Limosilactobacillus* and *Ligilactobacillus* (Figures 2, 3). Nevertheless, mechanistic resolution across the literature remains uneven. A limited number of bacteriocins, such as bactofencin A, have been investigated using structure-function analysis (Bédard et al., 2019) or stronger molecular characterization (O'Shea et al., 2013), whereas many others are still supported mainly by activity assays, estimated molecular masses, or microscopy-based observations (Mahdi et al., 2016, 2019). Future studies should therefore combine purification, quantitative activity testing, membrane assays, target identification, mutant analysis, heterologous expression, and standardized comparisons across indicator organisms. Such work will be essential for distinguishing shared bacteriocin principles from molecule-specific mechanisms and for linking antimicrobial activity more convincingly to translational potential.

In addition to direct antimicrobial effects, some bacteriocin-producing *Limosilactobacillus* and *Ligilactobacillus* strains have been reported to influence pathogen exclusion, microbiota composition, and host-associated immune responses (Mahdi et al., 2016, 2019); however, these effects should be interpreted as strain-level or indirect phenomena unless bacteriocin-specific evidence is available.

## Physicochemical robustness and functional stability

In addition to modes of antimicrobial activity of bacteriocins, their physicochemical characteristics determines sustenance of inhibitory activity when subjected to harsh conditions of food systems or therapeutic applications (Bali et al., 2016; Gharsallaoui et al., 2016). Several *Limosilactobacillus* and *Ligilactobacillus*-derived bacteriocins display substantial thermostability (Bacteriocin L23, fermencin SA715, reutericin 6, Bacteriocin TSU4) (Toba et al., 1991; Pascual et al., 2008; Sahoo et al., 2015; Wayah and Philip, 2018a), retain inhibitory activity across wide ranges of pH (Salivaricin mmaye1, Bacteriocin TSU4, Sal K and Alb β) (Pilasombut et al., 2005; Sangtanoo et al., 2014; Sahoo et al., 2015; Wayah and Philip, 2018b) and chemical stressors (Salivaricin mmaye1, Agilicin C7) (Wayah and Philip, 2018b; Yoo et al., 2023), but loose bioactivity in the presence of proteolytic enzymes (Bacteriocin L23, Fermencin SA715, Reutericin 6, Agilicin C7) (Toba et al., 1991; Pascual et al., 2008; Wayah and Philip, 2018a; Yoo et al., 2023), proving their proteinaceous attribute (Figure 1E). Structural characteristics such as stabilizing disulfide linkages, compact size, and cationic charge significantly contribute to their outstanding physicochemical attributes. These features, mechanistically explains why some bacteriocins tolerate harsh environments and justify their evaluation for various applications (Fahim et al., 2017; Liang et al., 2025).

## From antimicrobial activity to food biopreservation

One of the near-term promising applications of bacteriocins from *Limosilactobacillus* and *Ligilactobacillus* is Food preservation. Several reported bacteriocins or bacteriocin-containing preparations inhibit foodborne pathogens or spoilage organisms and have been tested in food-related matrices. Such systems include fruit (Wayah and Philip, 2018a), meat (Ying et al., 2023, 2025), fish (Shan et al., 2023), and bakery fillings (Therdtatha et al., 2016) (Table 1). Nevertheless, research have shown varying degrees of evidence strength. Bacteriocins, either fully or partially purified, are used in certain applications. In others, cell-free supernatants, crude fermentates, or protective cultures that produce bacteriocins are used. However, it is important to note that regulatory ramifications, repeatability, sensory consequences, and mechanical interpretability vary among the different formats.

Besides spectrum of antimicrobial activity, several other factors determine whether or not a bacteriocin will be effective in preserving foods. Such include pH level, the amount of salt, fats, proteins, storage temperature, packing conditions, and the food microbiota (Peng et al., 2023). The fact that a bacteriocin displays inhibitory activity in broth does not mean that the same activity will be seen when used foods. This is because foods can interfere with antimicrobial activity of bacteriocins. This happens because of breakdown by food proteases, unequal diffusion, binding of bacteriocin to food components, and reduced access to target cells (Peng et al., 2023; Putri et al., 2024). However, food processing conditions such as refrigeration, mild heating, high pressure, cold plasma or membrane-disrupting treatments might increase bacteriocin activity (Prudêncio et al., 2015), notably against Gram-negative bacteria, which are usually resistant due to their outer membrane.

Several examples have revealed that the behavior of bacteriocins is dependent on the application. Fermencin SA715 has been evaluated for preservation of fresh banana (Wayah and Philip, 2018a), while bacteriocin XJS01 has been tested in meat systems (Ying et al., 2023; Ng et al., 2025) and Lactocin-CM2 from have been combined with non-thermal plasma for aquatic food preservation (Shan et al., 2023). Additionally, bacteriocin -containing preparations from *Ligilactobacillus salivarius* KL-D4 slows down spoilage of creamy fillings (Therdtatha et al., 2016). These studies support the potential of bacteriocins as part of hurdle-preservation strategies. However, they should not be interpreted as evidence that all bacteriocins from these genera are broadly effective food preservatives. Each candidate requires validation in the specific food matrix, target organism, storage condition, and formulation in which it will be used.

Future food-application studies should therefore include matrix-specific testing, dose-response analysis, comparison with existing preservatives, sensory evaluation, stability during storage, and clear distinction between purified bacteriocin, partially purified preparation, crude fermentate, and live protective culture. Such standardization would make it easier to identify which bacteriocins are genuinely application-ready and which remain promising but preliminary candidates.

## Production optimization and scalability constraints

Translation of laboratory findings into commercial, medical and food applications needs the development of effective bacteriocin production systems (Abbasiliasi et al., 2017). In both *Limosilactobacillus* and *Ligilactobacillus*, bacteriocin biosynthesis is strongly associated with growth phase (Yan and Lee, 1997; Busarcevic et al., 2008; Wannun et al., 2016), regulatory mechanism (Wayah and Philip, 2018b), and composition of media (Vera Pingitore et al, 2009, Wayah and Philip, 2018b). For example, production of fermencin SA715 is enhanced in a new medium called SGSL in which manganese (II) sulfate is a critical requirement for its production. Other important nutrients that enhance its production include sodium citrate Tween 80, ascorbic acid, sodium chloride, magnesium sulfate, and sucrose. However, sodium chloride higher that 1% reduces the production of fermencin SA715. Also, optimum fermencin SA715 production occurs at 37 °C and pH 6.5 (Wayah and Philip, 2018a). Further, *Limosilactobacillus* and *Ligilactobacillus-*derived bacteriocin production peaks in the late exponential or stationary phase as is the case with fermencin SD11 (Wannun et al., 2016), reutericin 6 (Toba et al., 1991; Kabuki et al., 1997), bacteriocin LF-BZ532 (Rasheed et al., 2020), agilicin C7 (Yoo et al., 2023), and bacteriocin AU06 (Elayaraja et al., 2014). Production data across reported bacteriocins indicate that yield is not a fixed genus-level property but a strain-, peptide-, and medium-dependent phenotype. For example, some bacteriocins are produced maximally during late exponential (Salivarius CRL 1328) (Ocaña et al., 1999) or stationary phase (Bacteriocin LF-BZ532, Agilicin C7) (Rasheed et al., 2020; Yoo et al., 2023), whereas others require specific medium components (Salivaricin CRL 1328, Salivaricin KLD) (Vera Pingitore et al, 2009, Juárez Tomás et al., 2010; Therdtatha et al., 2016), pH values (Fermencin SD11) (Wannun et al., 2016), or divalent cations (Fermencin SA715) (Wayah and Philip, 2018a). This diversity implies that methods of optimization developed for one bacteriocin cannot be immediately applied to another, even within the same genus. Future production studies should thus provide standardized growth curves, activity units, peptide yield, medium composition, pH, temperature and purification recovery. This will allow comparisons of production efficiency of bacteriocins and producer strains.

Although some bacteriocins from these genera have been produced using optimized media (Fermencin SA715) (Wayah and Philip, 2018a) or heterologous expression systems (Bactofencin A) (Mesa-Pereira et al., 2017), scalable production remains inconsistent. Bactofencin A, for example, has been functionally expressed in *Escherichia coli*. This results show how heterologous systems can support production of selected bacteriocins (Mesa-Pereira et al., 2017). Nevertheless, the results obtained cannot be extended to other *Limosilactobacillus* and *Ligilactobacillus* bacteriocins. This is because expression, processing, secretion, immunity and peptide toxicity varied among bacteriocin types (Zimina et al., 2020; Tian et al., 2026). Scalable production will therefore require case-specific optimization of host strain, expression system, precursor processing, immunity protection, fermentation conditions, and downstream recovery.

## Purification bottlenecks and downstream limitations

Downstream processing remains one of the main obstacles to broader application of bacteriocins from *Limosilactobacillus* and *Ligilactobacillus*. Most reported workflows rely on multiple purification steps, including ammonium sulfate precipitation, dialysis, cation-exchange chromatography, hydrophobic-interaction chromatography, reverse-phase HPLC, and fast protein liquid chromatography (O'Shea et al., 2013; Mahdi et al., 2016, 2019). These methods are appropriate for purifying and characterizing bacteriocins in the laboratory but they are often difficult to scale. Reasons being that they can be labor-intensive, costly, time-consuming, and can cause substantial loss of antimicrobial activity (Jamaluddin et al., 2018).

The purification challenge differs among bacteriocins. Small hydrophobic peptides may require organic-solvent extraction or reverse-phase chromatography, whereas larger proteinaceous bacteriocins may require different capture and polishing strategies. For example, reutericin 6 has been purified using hydrophobic-interaction chromatography (Kabuki et al., 1997) and reverse-phase HPLC (Kawai et al., 2001; Arakawa et al., 2010). Also, in order to purify salivaricin SMXD51, bacteriocin LS1, and bacteriocin OR-7, precipitation, dialysis, ion-exchange, hydrophobic-interaction, FPLC, or reverse-phase chromatography were used (Stern et al., 2006; Busarcevic et al., 2008; Messaoudi et al., 2012). These examples show that purification processes are usually unique to each molecule. So, it should not be expected to be appropriate for all bacteriocins.

Another challenge is that in some studies, bacteriocins were only partially purified. Also, some did not provide information on how much of the bacteriocin was recovered, purity, yield or specific activity (Messaoudi et al., 2012; Wayah and Philip, 2018a; Sevillano et al., 2023; Halder and Mandal, 2025). Because of these reasons, it is difficult to compare different bacteriocins or determine feasibility of commercial production. In order to facilitate translation, it is important that future studies should report the starting activity in culture supernatant, activity after each purification step, final peptide yield, fold purification, percentage recovery, purity assessment, and specific activity. These parameters are essential for distinguishing bacteriocins that are promising only at laboratory scale from those that may be feasible for food, veterinary, or therapeutic applications.

Commercial production of bacteriocins requires development of simple methods of purification (Ng et al., 2025). Possible options are adsorption-desorption recovery from producer cultures, membrane-based concentration, expanded-bed adsorption, and food-grade precipitation methods. Additionally, research should focus on affinity-tagged heterologous expression for research-scale production, and engineered biofactories designed to secrete mature bacteriocins directly into the culture medium (Kaškoniene et al., 2017; Zou et al., 2018; Verma et al., 2022). If safety, repeatability, sensory impact and regulatory criteria are met, crude or semi-pure fermentates may be more feasible than highly purified peptides for food applications. In contrast, therapeutic applications will require stricter purity, identity, toxicity, immunogenicity, and batch-consistency standards.

Overall, purification should be considered early in the bacteriocin-discovery pipeline rather than after antimicrobial activity has already been demonstrated. A candidate with excellent *in vitro* activity but poor recovery, low stability during processing, or high purification cost may be less suitable for translation than a moderately active bacteriocin that can be produced and recovered reproducibly at scale.

## Challenges and future directions

### Translational bottlenecks in bacteriocin development

Despite the antimicrobial potential of bacteriocins from *Limosilactobacillus* and *Ligilactobacillus*, translation remains limited by gaps between discovery, characterization, production, and application testing. The reviewed bacteriocins differ markedly in the strength of evidence supporting their proposed uses. Some have been purified (Flynn et al., 2002; Wayah and Philip, 2018a), structurally characterized (O'Shea et al., 2013), linked to biosynthetic gene clusters (Flynn et al., 2002), or tested in food or animal models (Li et al., 2021). Others are supported mainly by inhibition zones (Mahdi et al., 2016; Yoo et al., 2023), cell-free supernatant activity (Abramov et al., 2022, 2023), partial purification (Mahdi et al., 2016), or molecular-mass estimation (Pascual et al., 2008). These differences should be considered when assigning translational potential.

One major issue is that results for antimicrobial activity are typically presented from laboratory settings that do not represent real-world scenarios. Biopreservation of food requires bacteriocins to maintain their activity in complex matrices that include microbiota, proteins, lipids, salts, and enzymes (Gálvez et al., 2007; Silva et al., 2018). They also must remain active throughout storage, processing, packing, and pH or temperature variations (Cotter et al., 2005). Other hurdles to therapeutic use include proteolytic breakdown, tissue distribution, toxicity, immunogenicity, microbiome effects, resistance development and transport to the site of infection (Mahlapuu et al., 2016; Sugrue et al., 2024). Therefore, it is important to view *in vitro* activity against an indicator strain as only a screening result and not as proof of application suitability.

Another recurring issue is the absence of standardized activity reporting. Because of variations in units, indicator organisms, assay settings, purification levels, and objectives (Pascual et al., 2008; Shan et al., 2020; Li et al., 2021; Halder and Mandal, 2025), direct comparison between studies is extremely challenging. Future studies should include MIC (minimum inhibitor concentration) or minimum bactericidal concentration where possible, clearly define activity units, report assay pH and matrix conditions, identify whether the test material is purified or partially purified, and include appropriate controls for acid, hydrogen peroxide, organic solvents, and other non-bacteriocin inhibitory factors.

The translational pipeline should therefore move from discovery to validation in a staged manner: identification of the producer and current taxonomy; confirmation of bacteriocin nature through protease sensitivity and purification; sequence or mass determination; biosynthetic gene-cluster analysis; antimicrobial spectrum and MIC testing; mechanism-of-action studies; stability and toxicity assessment; food-model or *in vivo* testing; and finally production, formulation, and regulatory evaluation. This staged approach would help prevent overinterpretation of preliminary antimicrobial activity and would allow stronger prioritization of candidates with realistic application potential.

### Regulatory and safety considerations

Future applications of bacteriocins from *Limosilactobacillus* and *Ligilactobacillus* will depend not only on antimicrobial potency but also on safety, regulatory classification, production consistency, and intended use (O'Connor et al., 2020). Regulatory expectations differ substantially for food preservatives, live biotherapeutic strains, veterinary products, topical antimicrobials, and systemic therapeutics (Cotter et al., 2013; O'Connor et al., 2020). So, safety evidence obtained for one application may not be appropriate for another.

For food applications, several things must be considered. These are, the safety status of the producing organism, absence of transferable antimicrobial-resistance genes or virulence determinants, reproducibility of bacteriocin production, activity in the intended food matrix, sensory impact, and stability during processing and storage (Cotter et al., 2005). For therapeutic or veterinary applications, more criteria have to be met. Areas that should checked include defined purity and identity, cytotoxicity testing, immunogenicity assessment, pharmacokinetic and pharmacodynamic evaluation, effect on microbiota, resistance monitoring, and exhibition of effectiveness in suitable models of infection (Cotter et al., 2013; Mahlapuu et al., 2016; Mookherjee et al., 2020).

Existing evidence suggests that some bacteriocins or bacteriocin-producing strains from these genera have favorable preliminary safety profiles. For example, bacteriocin TSU4 from *Ligilactobacillus animalis* (formerly called *Lactobacillus animalis*) TSU4 has been reported to be non-toxic and non-immunogenic in a mouse model (Sahoo et al., 2015, 2017), although reports are few, they provide a glimpse into the safety profiles of bacteriocins from this genera. For bacteriocins within *Limosilactobacillus* and *Ligilactobacillus*, safety conclusions should be made on a case-by-case basis and should distinguish purified-peptide safety from the safety of live producer strains.

Resistance development also requires more systematic evaluation. The narrow targets and membrane-associated mechanisms of bacteriocins are thought to make them less likely to cause resistance than conventional antibiotics. However, resistance can still develop due to changes in receptors, membrane composition, cell-envelope charge, immune systems, proteolytic degradation, or stress-response pathways (Draper et al., 2015; Sugrue et al., 2024). Importantly, resistance may carry fitness costs, but this should be experimentally demonstrated rather than assumed. Future safety assessment should therefore include serial-passage experiments, cross-resistance testing with clinically relevant antibiotics where appropriate, genome sequencing of resistant mutants, and evaluation of whether resistance alters virulence or ecological fitness.

Overall, for regulators to accept the product, there will need to be standard evidence packages that show molecular identity, production consistency, antimicrobial activity, safety, and environmental effect. This is especially important for bacteriocins that are meant to be microbiome-sparing antimicrobials, since protecting helpful microbial communities may be a part of their safety and effectiveness.

### Microbiome-sparing antimicrobial activity and ecological safety

**Figure 4:**
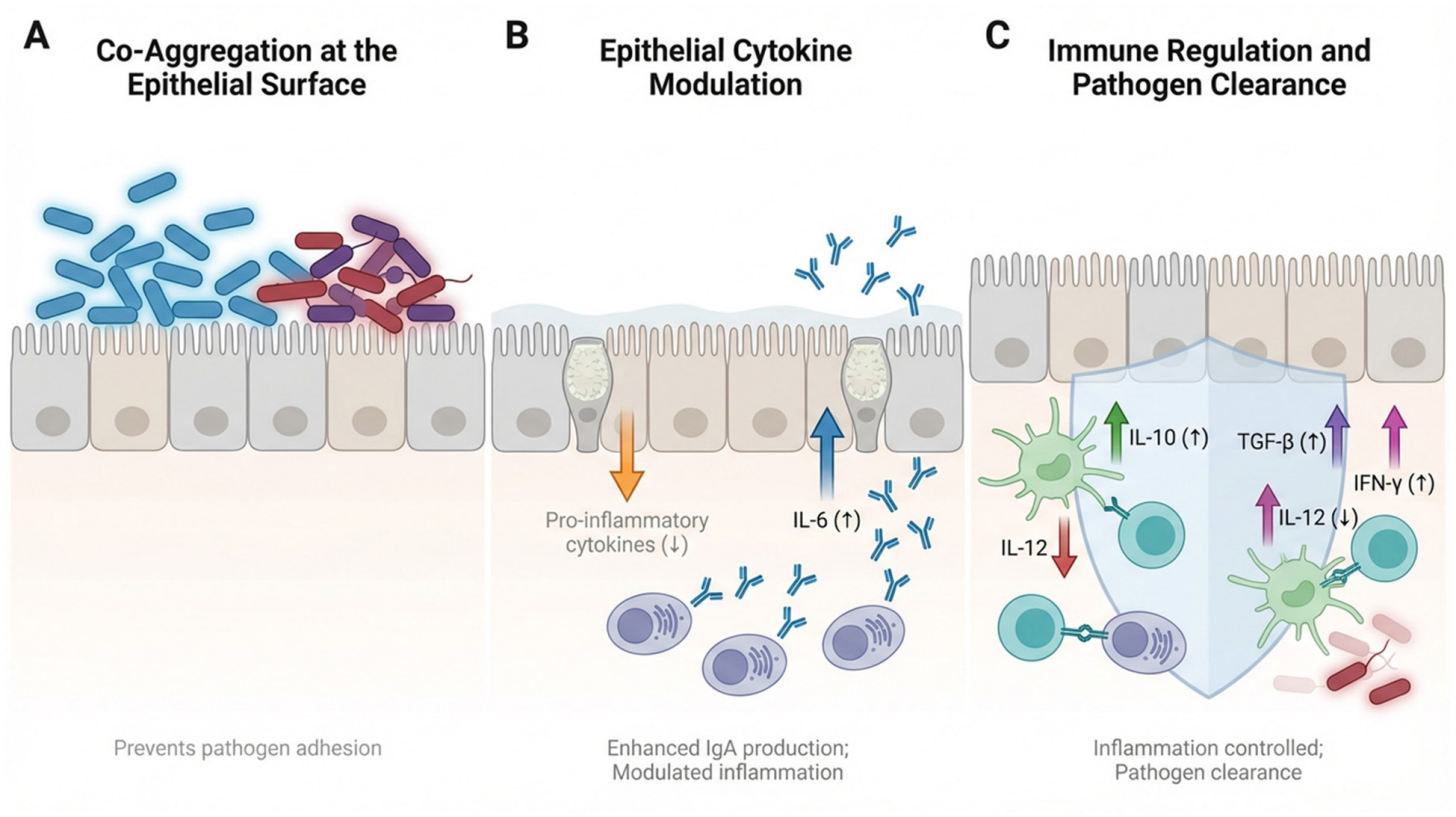
Host-interaction and immunomodulatory effects associated with bacteriocin-producing *Limosilactobacillus* and *Ligilactobacillus* strains. The effects shown should not be interpreted as bacteriocin-specific unless purified bacteriocins, bacteriocin-deficient mutants, complementation, or heterologous expression experiments support that conclusion. **(A)** Co-aggregation or competitive exclusion of pathogens by bacteriocin-producing strains, reducing pathogen adhesion to epithelial cells. **(B)** Modulation of epithelial cytokine responses, including altered pro-inflammatory signaling and possible stimulation of IgA-associated responses. **(C)** Immune regulation involving dendritic-cell and T-cell-associated pathways, including IL-10, IL-12, TGF-beta, and IFN-gamma signaling. These effects may reflect whole-strain probiotic activity rather than direct bacteriocin activity.

Before a bacteriocin can be considered for food and medical applications, it is important to examine how it impacts the microbiota of its host (Hernández-González et al., 2021; Bisht et al., 2025). Compared with broad-spectrum antibiotics, bacteriocins are often regarded as microbiome-sparing or precision antimicrobials. This is due to the fact that many display relatively narrow spectrum of antimicrobial action. This means that they kill or suppress the growth of microorganisms closely related to or share the same niche with their producer (Heilbronner et al., 2021; Nayak et al., 2022). This concept is particularly relevant for bacteriocins produced by *Limosilactobacillus* and *Ligilactobacillus*. This is so because many strains occupy densely populated host-associated niches. This includes the gastrointestinal tract, oral cavity, vagina, milk, and poultry environments. However, microbiome-sparing activity should not be assumed for all bacteriocins from these genera and must be demonstrated experimentally rather than inferred from taxonomic origin alone (Figures 1B, 4).

A reasonable way to examine how these bacteriocins impact microbiota of their host is by differentiating effects at the strain level from that at the bacteriocin level. In many studies, this impact was measured using live bacteriocin-producing strains (Abramov et al., 2022, 2023), crude culture preparations, or complex supernatants rather than purified bacteriocins (Mahdi et al., 2016; Elnar et al., 2025). Although these studies observed changes in colonization by pathogen, co-aggregation, epithelial signaling or composition of the microbiota this should not be attributed entirely to bacteriocin. Because acidification, exopolysaccharides, hydrogen peroxide, competition for nutrients, adhesion, and other probiotic ecological attributes may have contributed to the observations. Therefore, unless purified bacteriocins, bacteriocin-deficient mutants, complementation systems, or heterologous expression experiments are used, these findings should be interpreted as properties of bacteriocin-producing strains rather than as direct activities of bacteriocin molecules (Figure 4).

Available *in vivo* evidence remains limited but informative. Abp118, produced by *Ligilactobacillus salivarius* UCC118, did not significantly change the proportions of major groups of bacteria in the intestinal microflora of mouse and pig. This result supports the idea that some bacteriocin systems can act selectively without causing broad disruption of microbiota Riboulet-Bisson et al, 2012. Additionally, it has been observed that bactofencin A only subtly modulated populations gut of microorganisms. It did not restructure the entire community (Guinane et al., 2016). Research like this provides vital support for the idea that some bacteriocins or bacteriocin-producing strains can inhibit certain pathogens while maintaining the structural integrity of the microbiome around them. Nevertheless, the number of well-controlled *in vivo* investigations is small. Also, available evidence is not yet adequate to support a general conclusion that all bacteriocins from *Limosilactobacillus* and *Ligilactobacillus* display microbiome-sparing attribute across different hosts, delivery routes, or ecological environments (Figure 1B).

Ecological safety of bacteriocins entails more than how they impact make-up of a community. It also includes host-associated interactions. These interactions include pathogen exclusion, co-aggregation, and immunomodulation (Teng et al., 2023; Gu et al., 2024). Some bacteriocin-producing *Limosilactobacillus* and *Ligilactobacillus* strains (Bacteriocin SMXD51-producing Ligi*lactobacillus salivarius*, Salivirin LHM producing-Ligi*lactobacillus salivarius*, BLF3872 producing*-Limosilactobacillus fermentum*, Reutericin_LHS producing*-Lactobacillus reuteri*) (Mahdi et al., 2016, 2019; Saint-Cyr et al., 2017; Abramov et al., 2022) have been reported to reduce pathogen establishment by competing for adhesion sites, co-aggregating with pathogens, or influencing mucosal signaling pathways (Figures 4A–C). These observations are relevant to understanding how bacteriocinogenic strains may contribute to colonization resistance and host protection. However, as with microbiome effects, such findings are often demonstrated at the strain level and should not be attributed directly to the bacteriocin unless bacteriocin-specific evidence is available. Immunomodulatory responses, in particular, are likely to reflect integrated host recognition of the entire microorganism and its products rather than the peptide alone.

From a translational perspective, ecological safety should be assessed according to the intended product format. If the goal is to develop a purified bacteriocin, then microbiota impact, immunological effects, and safety must be demonstrated for the peptide itself. If the goal is to use a live bacteriocin-producing probiotic or protective culture, then the relevant biological unit is the whole strain, and bacteriocin production should be considered one component of a broader functional phenotype. Future work should therefore combine longitudinal microbiome analysis (Lawrence et al., 2022), defined *in vivo* models, strain-versus-peptide comparisons, and genetic validation Riboulet-Bisson et al, 2012 to determine when microbiome preservation and host-associated benefits can genuinely be assigned to bacteriocin activity.

Overall, the concept of microbiome-sparing antimicrobial activity remains one of the most promising translational features of bacteriocins from *Limosilactobacillus* and *Ligilactobacillus*. At present, however, the evidence supports this idea more strongly for selected bacteriocin systems than for the two genera as a whole. A more rigorous distinction between direct bacteriocin effects and broader strain-mediated ecological effects will be essential for evaluating both efficacy and safety in future food, veterinary, and therapeutic applications.

### Integrating ecological insight into rational bacteriocin discovery

Aligning strategies of bacteriocin discovery with ecological context is a key opportunity for moving the frontiers of bacteriocin research forward (Riley and Wertz, 2002). *Limosilactobacillus* and *Ligilactobacillus* occupy ecological niches such as the gut. These areas are often densely populated by microorganisms. Consequently, there is strong competition for nutrients which favors narrow-acting bacteriocins with action that is context-dependent (George et al., 2018; Mingolelli et al., 2025). It is pertinent to leverage this understanding to develop targeted screening strategies focused on bacteriocins adapted to specific pathogenic microorganisms. These ecology-driven discovery approaches may increase rate of success and decrease redundancy, propelling research on bacteriocins from wide exploratory investigations to rational, niche-informed unraveling of novel bacteriocins. However, in the absence of predictive frameworks that can integrate sequence, structure and ecological context, converting ecological concepts into discovery workflows that are scalable will remain difficult.

### Computational and AI-assisted approaches in bacteriocin discovery and optimization

The increasing availability of genome and metagenome sequences has expanded opportunities for bacteriocin discovery from *Limosilactobacillus, Ligilactobacillus*, and related lactic acid bacteria (Hemmerling and Piel, 2022). Computational approaches are useful because bacteriocin genes are often organized in biosynthetic gene clusters that may include genes encoding the precursor peptide, modification enzymes, transporters, immunity proteins, and regulatory elements (Lahiri et al., 2022). Tools such as BAGEL and antiSMASH can assist in detecting candidate bacteriocin gene clusters (Chou et al., 2026), while curated antimicrobial peptide and bacteriocin databases such as BACTIBASE (Hammami et al., 2010), DRAMP (Shi et al., 2022), APD3 (Wang et al., 2016), and CAMPR (Gawde et al., 2023) provide reference datasets for sequence comparison and functional prediction (Table 2).

**Table 2:** Representative computational and AI-assisted tools used in bacteriocin discovery and characterization, with their main applications and limitations.

Tool or resource	Main use in bacteriocin research	Limitation	References
BAGEL	Detection of bacteriocin gene clusters	Depends on known motifs and curated references	Egan et al., 2018; Sugrue et al., 2024
antiSMASH	Biosynthetic gene-cluster prediction	Broader natural-product focus; may miss atypical bacteriocins	Standiford, 2017; Sugrue et al., 2024
BACTIBASE	Curated bacteriocin reference data	Coverage may lag behind recent discoveries	Hammami et al., 2010; Akhter and Miller, 2024
DRAMP/APD/CAMPR	Antimicrobial peptide comparison and prediction	Not specific to bacteriocins from these two genera	Gabere and Noble, 2017; Ramazi et al., 2022
Macrel/AMPlify/AMPScanner-type tools	AMP prediction from sequence or metagenomic data	False positives require experimental validation	Veltri et al., 2018; Santos-Júnior et al., 2020; Xu et al., 2021; Li et al., 2022

Machine-learning and deep-learning approaches can be used to explore potential antimicrobial peptides. This may be achieved by predicting their activity (Cui et al., 2024), physicochemical properties, toxicity (David et al., 2021), structural features, and sequence-function relationships (Yan et al., 2022; Bonifacio-Velez de Villa et al., 2025). These methods work well for looking through big genome and metagenome datasets. In addition, they are useful for finding hidden peptides, and suggesting changes to the sequence that might make the compound more effective, stable, or easy to make (Saleem et al., 2025). But for now, their use for *Limosilactobacillus* and *Ligilactobacillus* bacteriocins is still more of an aid than a certainty. Training datasets are small, skewed toward bacteriocins that have been explored a lot, and not well annotated (Meng, 2025; Chou et al., 2026). Short cationic peptides might be mistakenly thought to be antimicrobial, and real bacteriocins might be missed if genes for defense, transport, or modification are not well labeled (Morton et al., 2015; Wan et al., 2024).

Consequently, experimental validation should be a part of AI-assisted discovery. It is recommended to test potential bacteriocins by looking at gene clusters, expressing or purifying peptides, testing for proteases, and testing for antimicrobial spectrum. Also, it is advised to determine MIC or activity units, testing for cytotoxicity, stability, and, if possible, genetic confirmation using knockout, complementation, or heterologous expression. For bacteriocins from *Limosilactobacillus* and *Ligilactobacillus*, the strongest future pipelines will combine taxonomic information, ecological source, gene-cluster architecture, predicted peptide properties, and application-specific validation. This integrated strategy would reduce false positives and help identify candidates that are not only antimicrobial *in vitro* but also suitable for food, veterinary, or therapeutic development.

### Toward scalable and application-ready bacteriocins

Advancement in bacteriocin research depends on progress in the fields of engineering, computation and biology. Synthetic biology may be able to raise the yield of bacteriocin by making production more efficient (Gabant and Borrero, 2019), enhance regulatory control (Todorov et al., 2022) and heterologous biofactories (Rosenkilde et al., 2024) (Figure 1C). Further, development of simple purification methods would help in commercialization of bacteriocins (Ng et al., 2025). Physicochemical properties (Field et al., 2018), antimicrobial activity (Field et al., 2015), and specificity (Field et al., 2015) of bacteriocins can be improved by spray-drying (Lenz et al., 2025), nanoencapsulation (Mohanty et al., 2025), and fusion with other antimicrobials peptides (Acuña et al., 2011; Arbulu et al., 2019). Also, genetic engineering can be used to produce genetically engineered *Limosilactobacillus* and *Ligilactobacillus* with enhanced capacity to produce bacteriocins which may be useful in preventing or treating infections of the gut (Carolak et al., 2025). Moreover, employing discovery pipelines involving computational tools and ecologic logic could greatly facilitate identification of bacteriocins that possess high antimicrobial activity and are inherently suitable to settings where they will be applied. By employing these strategies, bacteriocins can transition from promising antimicrobials to scalable solutions for food, veterinary and therapeutic applications.

## Conclusion

The 2020 reclassification of the former broad *Lactobacillus* genus provides a useful framework for re-examining bacteriocin diversity in a more precise taxonomic and ecological context. Within this framework, *Limosilactobacillus* and *Ligilactobacillus* represent two informative host- and food-associated genera that include multiple reported bacteriocin-producing strains. Their bacteriocins span different structural classes, genetic organizations, antimicrobial spectra, and levels of experimental characterization.

The main value of examining these genera is not that their bacteriocins possess features absent from other lactic acid bacteria. Many properties discussed in this review, including membrane activity, narrow or selective inhibition, protease sensitivity, thermostability, quorum-associated regulation, and food-preservation potential, are shared by bacteriocins from other taxa. Rather, the value lies in linking these features to current taxonomy, ecological niche, strain origin, biosynthetic organization, and translational evidence. Using this method is helpful with regards differentiating observations made at the genus level from those specific to bacteriocin. Consequently, this helps in preventing broad application of findings from individual studies.

Currently available information suggests that certain bacteriocins from *Limosilactobacillus* and *Ligilactobacillus* could be used as antimicrobials in food, medicine, and animals. The proof base is still not uniform, though. Some of the bacteriocins have been purified, structurally characterized, genetically linked to biosynthetic gene clusters, and tested in food or *in vivo* models. However, observations from some bacteriocins are based on basic inhibition tests or partly purified preparations. So, in the future, researchers should focus on standardized taxonomy, and distinguish between purified bacteriocin and producer-strain effects. Also, attention should be given to quantitative activity testing, mechanism validation, safety assessment, microbiota-impact studies, scalable production, and development of simple purification methods.

Computational and AI-aided methods can speed up the search for and ranking of potential bacteriocins. However, they cannot replace validation through experiments. Genome mining, ecological source information, peptide prediction, analysis of biosynthetic gene-cluster, purification, mechanistic examination, and application-specific validation will all be part of the most productive system of the future. This kind of unified approach will make it easier to test bacteriocins from *Limosilactobacillus, Ligilactobacillus*, and other lactic acid bacteria more thoroughly as precise antimicrobials and biopreservatives in the post-*Lactobacillus* era.
